# Medical case reports in the age of genomic medicine

**DOI:** 10.1038/cti.2015.15

**Published:** 2015-10-16

**Authors:** Matthew C Cook

**Affiliations:** 1 Department of Immunology, John Curtin School of Medical Research, Australian National University, Woden, Australian Capital Territory, Australia; 2 Translational Research Unit, Canberra Hospital, Woden, Australian Capital Territory, Australia

## Abstract

The case report has been a pillar of medical literature but has been displaced recently because of inherent risks of bias. As we move towards precision medicine, however, the case report format could provide an important method for describing disease mechanisms based on rare genetic variants. Empirical evidence reveals that many previously unexplained Mendelian diseases are accounted for by rare heterozygous alleles, *de novo* mutations or compound heterozygous mutations, and that disease-associated variants are often confined to the kindred of the affected individual. Elucidation of the phenotypes of these rare genetic variants will necessarily offer unique insights into disease mechanisms. Even when the association between variants in a specific gene and a disease has already been identified, individual cases are valuable. Allelic series extend both the clinical and laboratory phenotypes. Finally, the prevalence of a disease is not a reliable indicator of the therapeutic importance of the underlying mechanism, so resolving extreme phenotypes even in single cases has the potential to identify new treatment strategies relevant to more common disease.

## Overview of the medical case report

The case report is a detailed description of the experience of a single patient that often serves to illustrate a disease, a therapeutic intervention or the approach to a diagnostic dilemma. Typically, case reports take the form of a narrative of clinical experience, documenting the diagnostic process and treatment. In medical genetics, case reports have been used to identify unusual or distinct phenotypes, or indeed clusters of phenotypes that constitute novel syndromes that require explanation. To some extent, the heuristic value of the case report depends on the exceptional nature of the case, reflecting either the novel or unusual disease phenotype, or a putative association between two or more disease phenotypes, which might suggest a common aetiology for the concordant diseases. In addition, case reports often contain an element of surprise, because of information that seems at odds with prevailing models or assumptions,^1, 2^ which presents the reader with information that demands an explanation.

Traditionally, the case report has focused on the rare or unusual, the curious and exceptional. This might be seen as a virtue with the increasing recognition that rare diseases collectively account for a substantial proportion of human morbidity and mortality, and therefore they demand greater attention.^3^ On the other hand, it is also a reason for case reports falling out of favour recently. Another reason is concern about bias in case reports.

Proponents of evidence-based medicine (EBM) have relegated case reports to the lowest rank on the hierarchy of evidence on which clinical decisions are made. This places them somewhere near pathophysiological mechanism as a basis for clinical decision-making.^4, 5^ Instead, EBM places great emphasis on the comparative clinical study, usually using double-masking, placebo controls and large sample sizes. Biomedical researchers have also criticized the case report format because it is often a descriptive narrative, largely concerned with phenotype, and not experimental.

## Signs of life in the case report

Under some circumstances, case reports have been adapted to the aims of EBM, for example, to illustrate evidence-based practice in action by using a particular case to illustrate how to garner available evidence and bring it to bear on clinical decision-making. Furthermore, expert discussion of a challenging case in the grandrounds format remains acceptable, and popular, as illustrated in the case records of the Massachusetts General Hospital reported weekly in the *New England Journal of Medicine* and the *The Lancet* case reports. Overall, however, the case report as a method is at odds with EBM, which seeks to gather evidence that can be applied generally. EBM seeks to capture the typical and confirm what might already be in practice. Evidence is necessary to guide therapy, and this needs to be gathered from many individuals because of biological and analytical variation.

The randomized control trial seeks to minimize bias in trial design to identify effects of treatment that are greater than biological variation, and not due to the spurious action of unknown variables. Additional variation, however, arises from heterogeneity of patients included within the study sample because of limitations in the prevailing nosology. In other words, heterogeneity of response reflects in part our current state of ignorance about disease mechanisms, which prevents adequate case definition and disease stratification.^6^ For example, until recently non-small cell lung cancer was a histological and clinical diagnosis, but now, we know that approximately 5% of these patients harbour the *EML4-ALK* translocation, which makes them ideal candidates for targeted treatment.^7^

Until we can account for disease heterogeneity, large sample sizes will remain an important tool to assess the efficacy of therapy. Nevertheless, the failure of randomized control trial to demonstrate a therapeutic effect of certain drugs does not necessarily vindicate the decision of EBM to relegate therapy based on the knowledge of mechanism. Another explanation is our inability to identify the correct target group of patients for a particular therapy based on our current state of knowledge. This risk is large when the study cohort is defined according to end-organ damage rather than more proximal disease mechanisms (because this maximizes cohort heterogeneity), and when the trial drug is a precision medicine (because this is likely to require disease stratification according to mechanism to demonstrate a therapeutic effect).

## Genomic medicine

Genomic or personalized medicine is predicated on individual variation, and our ability to characterize variation between patients who have attracted a particular diagnosis by capturing comprehensive genotypic and phenotypic data (typically using techniques whose names with the suffix –omics, meaning for all).^8^ Characterization of genome-wide variation has started to penetrate clinical medicine, and this has already led to a change in medical ontology. As we start to practice genomic medicine, analysis and discovery based on rare cases is an obvious vehicle for reporting progress. While the randomized control trial is the epistemological tool for EBM, in some situations, the case report is appropriate to personalized medicine.

The traditional case report has served to either raise important questions or identify unusual associations or effects of treatment (beneficial or detrimental). The best case reports have assumed importance because the implications of the findings reported turn out to be both general and substantial.^9^ For example, identification of the Leiden allele of factor V in a family with thromboembolic disease.^10^

A case report might not only flag a more general phenomenon, but may also be an end in itself, because the disease phenotype that is explained might turn out to be extremely rare. Even individual cases of a genetic variant or different alleles of a gene already implicated in disease might prove to be exemplary if the case identifies a pathway that requires further explanation. After this, additional descriptions of single cases can inform the extent of genetic heterogeneity for a particular phenotype. When combined with mechanistic analysis, it sometimes emerges that the genetic heterogeneity reflects mutations affecting proteins within the same physiological pathway.

When analysed in depth, rare diseases represent natural experiments, and their elucidation can be of considerable importance in understanding complex disease mechanisms. This concept is exemplified by rare infection phenotypes such as herpes simplex encephalitis (Table 1). Individual case reports have identified this as a novel and specific disease arising from single gene disorders. Herpes simplex encephalitis is genetically heterogeneous, but so far, each variant has occurred in a gene encoding a protein involving either the innate immune sensor toll-like receptor 3 (TLR3), a regulator of its intracellular trafficking (UNC93B) or a component of the downstream signaling complex (TICAM1).^11, 12, 13^

## Case reports meet medical genomics in the age of genomic medicine

Almost 4000 extreme phenotypes arising from monogenic defects have been described (OMIM). Offspring of consanguineous parents or individuals from geographically isolated populations have provided an important way of identifying rare homozygous mutations identified in single families. Such cases have been elucidated by whole-exome sequencing and remain tremendously informative for understanding genotype–phenotype relations.^14^

The extent of genome variation from person to person provides the rationale for case reports involving individuals with severe sporadic disease or extreme phenotypes. Each individual carries about 3 × 10^6^ single nucleotide variations and more than 1000 copy number variations^15, 16^ when compared with the reference haploid genome. A substantial proportion of these (>2 × 10^5^) are private to the patient or their family.^17, 18^ Equally remarkable is the finding that a significant proportion of this genetic variation has arisen recently. Each haploid genome appears to harbour 100 or so coding genetic variants that have not been identified outside the kindred under investigation. Furthermore, each individual carries *de novo* genetic variants. The rate of *de novo* mutations is approximately ~2 × 10^−8^.^19, 20^ New copy number variations arising *de novo* are even more common.^21^ Neither recent rare private mutations nor *de novo* mutations have been subjected to purifying selection, and therefore are likely to include damaging variants. Collectively, these observations mean that extreme phenotypes may arise from rare genotypes. In the offspring of consanguineous parents, rare homozygous variants are expected, whereas *de novo* heterozygous, X-linked recessive or compound heterozygous mutations are plausible explanations for apparently sporadic disease in non-consanguineous families.

As the prevalence of rare damaging variants is surprisingly high, it follows that descriptions of rare cases are not just informative, but necessary in the era of PM. The validity of this approach has been demonstrated in diseases of immunity and summarized by Casanova and colleagues.^22^ By 2014, approximately 50 forms of immune deficiency had been reported in single cases.

Genomic medicine case reports demand that the genotype is a plausible explanation for the phenotype and therefore that the consequences of the mutation disrupt the protein function. For exceptional sporadic diseases, the phenotype is expected to be extreme or rare. To explain the phenotype of a single case, *de novo* mutations are strong candidates, especially when they affect a gene whose function is known, and could be reasonably implicated in the disease, based on the demonstrated cellular and biochemical phenotype. Similarly, private heterozygous mutations acting as autosomal dominant mutations are good candidates. Compound heterozygous mutations may not necessarily require two private mutations to emerge as candidates, but a private genotype might be expected.

The first case report of a new disease is important but so are follow-up reports, particularly if they constitute an allelic series, because this can provide confirmation that mutations in a particular gene cause a syndrome, or to extend the phenotypic disease spectrum associated with mutations in a particular gene. Indeed, such an approach might be valuable in identifying new therapeutic targets.^23^

Case reports in genomic medicine might not only be pertinent to rare phenotypes. Rare variants can explain more common disease phenotypes when the mutational target is large, that is, where mutations in many genes can result in a similar phenotype, such as intellectual disability. Under these circumstances, the combination of measurable *de novo* mutation rate and large mutational footprint can account for conditions that are lethal or associated with low rates of reproduction (that is, selection coefficient ~1.0).^17^ Case reports will eventually provide a catalogue that permits empirical determination of the size of the genetic footprint of such diseases.

Even when a variant in a specific gene has already been implicated in a particular disease, further cases are informative. A lesson from primary immune deficiency diseases is that there is considerable phenotypic variance even with monogenic diseases. Furthermore, the extent of phenotypic variance is often greatest at the level of clinical manifestations of signs and symptoms, whereas laboratory-determined phenotypes can be more consistent. In addition, there are now cases where phenotypic variance is accounted for by differences in the polarity of effect of the mutation, where both gain-of-function and loss-of-function variants have been described (Table 2).

## What is required from an informative case?

The case report has the potential to once again assume an important and prominent place in clinical medicine, but this will only be realized if certain high standards are adhered to in order to ensure that case reports represent progress, rather than the noise that EBM has worked so hard to overcome. As a tool of PM, the case would be expected to provide some support for a pathophysiological pathway, and therefore should encompass a nuanced report of the clinical phenotype and, where possible, a sophisticated account of the extended phenotype by detailed analysis of laboratory data, combined with a report of the genotype or indeed the patient genome and the informative variant. Ideally, the case report would provide evidence of a causal association between genotype and phenotype.

At present, a genomic medicine case report is most likely to concern a simple Mendelian disease, although as progress is made in understanding disease mechanisms, more complex cases might become the subject of exemplary reports. Some diseases thought to be monogenic will turn out to be oligogenic (that is, phenotypic variants that arise from gene–gene interactions).

The greatest value accrues from case reports that provide novel insights. Functional studies are expected to demonstrate causation of the variant allele, based on an animal model, or genetic complementation. The evidence required to establish causation from a single case can be hard to achieve. Nevertheless, opportunities exist, such as isolating the causative mutation from the remainder of the genome, including by replicating the mutation using genome-editing techniques such as CRISPR/Cas9.^24, 25^

One of the important functions of the case report has always been to stimulate other clinicians to reconsider cases they have seen in the light of the published findings and questions and hypotheses raised. As a result, this can lead to the identification of additional cases, which provide rapid progress and confirmation of the mechanism, or indeed, expansion of the phenotype associated with a particular gene variant. This goal should remain in the age of personalized medicine, where the case report should remain both informative and stimulating, and an example of the increasing possibility that clinical medicine and discovery can coalesce.

## Figures and Tables

**Table 1:** Monogenic causes of herpes simplex encephalitis reveal susceptibility pathway

*Gene*	*Inheritance*	*Cases*	*Genotypes*	*Reference*
*UNC93B1*	AR	2	2	^12^
*TLR3*	AD	2	2	^11^
*TRAF3*	AD	1	1	^13^
*TICAM1*	AD	1	1	^26^
*TICAM1*	AR	1	1	

**Table 2:** Examples of genes harbouring both loss- and gain-of-function mutations

*Gene*	*Phenotype*	*Inheritance*	*Cases*	*Genotypes*	*Reference*
*STAT1*					
Loss of function	Disseminated low virulence mycobacteria	AD	1	1	^27^
	Disseminated low virulence mycobacteria and herpes simplex encephalitis	AR	1	1	^28^
Gain of function	Chronic mucocutaneous candidiasis, invasive fungal infection, herpes virus infection and autoimmunity	AD	14	2	^29^
					
*STAT3*					
Loss of function	Job's syndrome. Recurrent *Staphylococcus aureus* infection and mucocutaneous candidiasis. Skeletal abnormalities	AD	15	8	^30^
Gain of function	Type 1 diabetes, encephalopathy, autoimmune thyroid disease	AD (*de novo*)	5	4	^31, 32, 33^
